# A cross-country analysis on diversification, Sukuk investment, and the performance of Islamic banking systems under the COVID-19 pandemic

**DOI:** 10.1016/j.heliyon.2022.e09106

**Published:** 2022-03-12

**Authors:** Tu DQ. Le, Tin H. Ho, Dat T. Nguyen, Thanh Ngo

**Affiliations:** a University of Economics and Law, Ho Chi Minh City, 700000, Vietnam; b Vietnam National University, Ho Chi Minh City, 700000, Vietnam; c Massey University, Palmerston North, 4414, New Zealand; d VNU University of Economics and Business, Hanoi, 10000, Vietnam

**Keywords:** Diversification, Sukuk investment, Islamic banking systems, Performance, COVID-19, Generalized method of moments (GMM)

## Abstract

This study investigates the relationship between diversification and Islamic banking systems' performance under the impact of the COVID-19 turmoil using a sample of 24 countries from 2013Q4 and 2020Q4. The findings indicate that the performance of Islamic banking systems is positively associated with sectoral diversification of Shari'ah-compliant financing and income diversification. Although this study confirms a negative impact of the COVID-19 shock, income diversification is found to mitigate the adverse effect of this health crisis on the performance of the Islamic banking systems. In which, Sukuk investment is considered an essential channel for pursuing this diversification strategy. Therefore, this research has important implications for policymakers, managers, and academics.

## 1 Introduction

It is acknowledged that the COVID-19 turmoil has challenged the global economy (International Monetary Fund, 2021b; McKibbin and Fernando, 2021). Due to the spread of the Coronavirus, governments worldwide have imposed several policy measures such as social distancing and lockdown. Therefore, this has affected the operation of the banking systems itself directly but also the performance of the banking systems indirectly via affecting the households' income and businesses' revenue. The adverse impact of the COVID-19 shock on the financial system is documented in the literature (e.g. Feyen et al., 2021; Gormsen and Koijen, 2020; International Monetary Fund, 2021a, 2021b; McKibbin and Fernando, 2021; Ramelli and Wagner, 2020). However, studies on the COVID-19 impacts on the banking system are still limited (Demirgüç-Kunt et al., 2021; Elnahass et al., 2021).

The global Islamic banking sector is undeniably becoming more critical (S&P Global Ratings, 2020); however, it is not exceptional under the impact of this pandemic. Indeed, the Islamic banking system plays a vital role in several jurisdictions where its share accounted for approximately 15% or more of the overall banking sector (IFSB, 2019). In these countries, the Islamic banking sector is one of the crucial sources of funding to the economy, particularly small enterprises (Islamic Development Bank, 2020). Prior to the COVID-19 pandemic, the Islamic banking system had experienced significant growth and stable projections - the Islamic Development Bank (2020) reported double-digit growth in total assets and financing of the Islamic banking systems from 2013 to 2018. The onset of the COVID-19 turmoil and the significant reduction in oil prices, however, have caused a dual shock for most Islamic banking systems in countries mainly based in oil-producing countries. This ultimately affects the Islamic banking sector regarding asset quality and profitability (as further explained in the later section). Therefore, the impacts of COVID-19 on the Islamic banking system have received much attention from academics, practitioners, and policymakers. Although Islamic banks are better to withstand endogenous risks (e.g., the Global Financial Crisis) (Beck et al., 2013; Bourkhis and Nabi, 2013; Tabash and Dhankar, 2014), this study strikes to answer whether the performance of the Islamic banking systems is significantly affected by the COVID-19 pandemic.

Furthermore, prior studies on conventional banking systems suggest that income diversification may improve bank profitability during this health crisis (Li et al., 2021). Similar to non-interest income for conventional banks, non-financing income (so-called income un-associated with deposit-taking and loan advancing) includes fee and commission income, trading income, and other non-financing income (Molyneux and Yip, 2013). Chen, Liang, and Yu (2018) noticed that the effect of diversification might differ between conventional and Islamic banking systems. Empirical evidence on the relationship between diversification and Islamic bank performance is mixed. Several studies support the benefits of diversification (Chatti et al., 2013; Chen et al., 2018; Molyneux and Yip, 2013). However, others show opposite findings (Paltrinieri et al., 2020). Due to the mixed results on diversification, we revisit this issue under the impact of global health crises.

This study contributes to the literature in two important ways. First, the evidence on the relationship between the COVID-19 pandemic and the Islamic banking system is scantier (Demirgüç-Kunt et al., 2021; Elnahass et al., 2021; Miah et al., 2021; Rehman et al., 2021). Those studies, however, looked at the Islamic banking sector via a micro-perspective using data from individual banks. Hence, a macro-perspective on the whole Islamic banking system is still missing. We contribute to that literature by using aggregate data of 24 Islamic banking systems countries to examine whether they were, as the whole systems, affected by the COVID-19 pandemic. Second, we investigate whether the diversification strategy may mitigate the adverse effects of the COVID-19 shock on the profitability of the Islamic banking systems. In this setting, we considered two measures of banking diversification, including sectoral diversification of the Shari'ah-compliant financing and income diversification. Regarding the rapid development of the Sukuk market (IFSB, 2020), we further examine whether Sukuk holdings in the portfolio of Islamic banking systems, as a part of income diversification, may reduce the negative impact of the COVID-19 turmoil. By doing so, the findings would shed light on the importance of diversification in the Islamic banking systems' performance during the COVID-19 period.

Using the national-level data of 24 countries between 2013Q4 and 2020Q4, our empirical findings show that the Islamic banking systems are negatively affected by the COVID-19 pandemic. This is consistent with the literature on the adverse impact of the COVID-19 turmoil on the conventional banking sector (Demirgüç-Kunt et al., 2021; Elnahass et al., 2021). Furthermore, the findings indicate the benefits of banking diversification on the profitability of the Islamic banking systems in general, either sectoral diversification of Shari'ah-compliant financing or income diversification. Additionally, only income diversification may alleviate the negative effect of the COVID-19 shock on the profitability of the Islamic banking system, especially Sukuk investments. Nonetheless, this reemphasizes the importance of a shift towards commission and fee income and non-financing activities in the profitability of the Islamic banking system.

The remainder of this study is organized as follows. Section 2 presents a brief literature review. Section 3 provides the methodology and data used in this research. Section 4 discusses empirical results, while Section 5 concludes.

## 2 Literature review

### 2.1 Diversification and Islamic banking systems’ performance

Islamic banks, like conventional banks, usually borrow money from depositors and lend it to borrowers, relying on the net interest margin to make profits. Apart from interest income, non-interest income, such as fees and commissions or trading, can also generate profit for the Islamic banking systems (Molyneux and Yip, 2013).

However, previous research using bank-level data shows that Islamic banks are less diverse than conventional banks and, hence, may benefit more from income diversification or asset diversification, which can also help to improve financial stability. Specifically, Molyneux and Yip (2013) demonstrate that non-financing revenue has a positive net overall impact on banks' risk-adjusted performance, resulting from increasing income volatility. In comparison to conventional banks, Islamic banks are more concentrated on traditional activities and less diverse in income sources. Similarly, Paltrinieri et al. (2020) recently suggest that income diversification provides lower rewards for Islamic banks than conventional banks, with the impacts being higher for accounting-based indicators than those of market-based. Non-interest income proportions contribute favourably to profitability regardless of the business model, but it has little influence on the risk-adjusted profitability of Islamic banks. Furthermore, neither conventional nor Islamic banks show any correlation between income diversification and bank stability. These findings are in line with previous studies (Abuzayed et al., 2018; AlKhouri and Arouri, 2019). On the other hand, Islamic banks' services required for specialized activities (for example, Sukuk underwriting) and religious concerns (e.g., Hajj), may enhance Islamic banks’ profitability.

Apart from income diversification, Shari'ah-compliance or sectoral diversification (including prohibitions on interest-based transactions, undue uncertainty, gambling, and among others) has a substantial impact on their business strategies. Simultaneously, adhering to Sharia, in addition to other legislative standards, may result in more significant expenses and the exclusion of some illegal commercial operations. As a result, the net advantages of diversification for Islamic banks might be reduced (Paltrinieri et al., 2020).

Considering the above observations, our research takes a step forward in unravelling such complicated linkages by examining whether income diversification and sectoral diversification impact the performance of Islamic banking systems. In general, the diversification degree of Islamic banks is relatively lower than conventional counterparts, and therefore allowing them to reap diversification benefits. Hence, the first two hypotheses are as follows.

H1 Income diversification has a significant effect on the Islamic banking systems' performance.

H2 Sectoral diversification has a significant effect on the Islamic banking systems' performance.

### 2.2 Sukuk investment and Islamic banking systems’ performance

The term “Sukuk” refers to certificates and is the plural form of the Arabic word “Sak”, which means “certificate” (Iqbal and Mirakhor, 2007). Sukuk includes financial securities issued by governments or enterprises to fund various projects, and they have grown in popularity over the last two decades (McMillen, 2007). Sukuk raises debt-like security with qualities similar to traditional bonds in terms of cash flow and risk. Sukuk, on the other hand, is akin to conventional debt/bonds with artificial variations. Indeed, many studies have emphasized the specific characteristics and reasoning for releasing Sukuk (Azmat et al., 2015; Mohamed et al., 2015; Nagano, 2017; Naifar et al., 2017). On top of that, Sukuk is not vulnerable to the financial crisis in 2008 or contagion risks during global shocks, providing foreign investors with a portfolio diversification option (Kenourgios et al., 2016; Naifar et al., 2017).

Despite an increase in worldwide Sukuk issuances and market size, existing research on Sukuk, one of the two pivotal pillars of Islamic finance along with Islamic banking, is still sparse and dispersed across several themes (Ibrahim, 2015), except for comparing the performance of Islamic banks to conventional banks (Beck et al., 2013; Bitar et al., 2018; Johnes et al., 2014; Olson and Zoubi, 2017; Safiullah, 2021). Smaoui, Mimouni, and Temimi (2017), for example, explore the interaction between the banking sector and Sukuk markets and demonstrate that banks and Sukuk are alternatives. Even though their analysis did not explain this substitution or whether conventional banks and Islamic banks compete equally with Sukuk, it serves as a starting point for a more in-depth look into the relationship between Sukuk markets and the banking sector. The connection between the bond market and the banking industry, and the well-documented cointegration link between Sukuk and conventional bonds, necessitates such an examination (Hassan et al., 2018).

A recent study related to this topic comes from Mimouni et al. (2019). Their analysis finds that the growth of Sukuk markets reduces Islamic banks' profitability as assessed by NIM (net interest margin) and NPM (net profit margin) or even ROA (Return on Asset), but has no impact on that of conventional banks. Smaoui and Ghouma (2020) similarly demonstrate that the development of the Sukuk market has had a detrimental effect on Islamic banks' capital ratios. They believe that the Sukuk growth has fuelled rivalry among Islamic banks, causing them to maintain lower capital levels. The mixed findings in Sukuk, a fast-developing instrument of financial markets, and Islamic banks’ performance encourage us to develop the third hypothesis.

H3 Sukuk investment has a significant effect on the Islamic banking systems' performance.

### 2.3 COVID-19 and the Islamic banking systems

In our study, special attention is devoted to the COVID-19 pandemic. Indeed, the existent literature documents that COVID-19 has had a negative influence on financial performance across different indices (i.e., accounting-based and market-based performance measures) and financial stability (i.e., default risk, liquidity risk) in the worldwide banking industry (Demirgüç-Kunt et al., 2021; Elnahass et al., 2021). These findings are similar across geographies, nations, and bank-level characteristics but different in financial systems (i.e., conventional and Islamic) (Elnahass et al., 2021). Likewise, Hasan et al. (2021) indicate that the pandemic creates identical volatility in conventional and Islamic banking stock markets. To explain the distinct effects between conventional and Islamic banks during crises, Beck et al. (2013) and Mollah et al. (2017) suggest that Islamic banks are more complicated than their conventional counterparts and were better shielded during the global financial crisis in 2007. Although differential impacts on bank stability and stock market values exist between the two bank types (Abdelsalam et al., 2020), it is uncertain whether these past findings will remain under the unprecedented COVID-19 shock, and whether Islamic banks would be sufficiently strong and resilient to prevent such upheaval. Given that the Islamic banking systems tend to be more resilience than their conventional banking counterparts, the fourth hypothesis of our study is:

H4 COVID-19 has a significant impact on the Islamic banking systems' performance.

Given the impact of the COVID-19 pandemic and diversification, this study further investigates the interaction effect of the COVID-19 shock and diversification on the performance of the Islamic banking systems. The following hypotheses are formed:

H5 Income diversification is likely to reduce the negative impact of the COVID-19 pandemic on the Islamic banking systems' performance.

H6 Sectoral diversification is likely to reduce the negative impact of the COVID-19 pandemic on the Islamic banking systems' performance.

As Sukuk investment is a part of income diversification, the joint effect of Sukuk investment and the COVID-19 turmoil on the performance of the Islamic banking systems is also examined.

H7 Sukuk investment is likely to reduce the negative impact of the COVID-19 pandemic on the Islamic banking systems' performance.

## 3 Methodology and data

### 3.1 Methodology

Prior studies using aggregate data to examine the determinants of banking performance suggest that a system generalized method of moments could overcome unobserved heterogeneity and endogenous problems (Le and Ngo, 2020; Le et al., 2020). Also, the persistence of banking performance is accounted for by this method, and thereby the system GMM can yield consistent estimations of the parameters (García-Herrero et al., 2009).

For instance of endogeneity issues, low efficient Islamic banking systems may be required to maintain a higher level of capital (Le, 2018, 2021). This relationship could go in the opposite direction. A greater level of bank capitalization may permit Islamic banking systems to make risky investments with greater returns. Given the higher level of bank risk, this may incur additional resources and organizational efforts to deal with it, and thus reducing the efficiency of the Islamic banking system. Additionally, diversification may affect bank capitalization positively and reduce bank efficiency (Rossi et al., 2009).

Following Bond (2002), the lagged values of the dependent and endogenous variables will be instrumented in the system GMM, except for exogenous regressors. It is assumed that endogenous variables are predetermined while exogenous variables are strictly associated with individual effects. Given that the system GMM is relatively sensitive to the number of lags, we follow Fu et al. (2015) and Le (2019) by using the one-year lagged values of all potentially endogenous regressors as instruments in our regressions. This is because the use of more lags would cause weak instruments. The results of the Arellano-Bond autocorrelation (AR) and the Hansen tests further confirm the use of the number of lags (Le et al., 2020).

The literature suggests that the performance of Islamic banks is affected by the Sukuk capital market. It is observed that the Sukuk holdings in Islamic banking systems may vary among countries included in our sample (as further discussed in the next section). First, using a more extended period to examine the effect of Sukuk development on the performance of the banking system, our baseline model is formed as follows:

(1) $${\text{π}}_{i,t}=\alpha_{0}+\alpha_{1}{\text{π}}_{i,t-1}+\alpha_{2}SUKU_{i,t}+\alpha_{3}DIV_{i,t}+\alpha_{4}X_{i,t}+\varepsilon_{i,t}$$

where bank performance (π) can be measured in two ways, including ROA, returns on assets; ROE, returns on equity. SUKU is measured by the value of Sukuk holdings to capital. Given that the quarterly data on the Sukuk issued by the Islamic banking systems were missing in many countries, we consider the effect of holding Sukuk on the profitability of the Islamic banking system. Banking diversification (DIV) can be proxied in two different ways: the sectoral diversification of Shari'ah-compliant financing (DIV−SEC), income diversification (DIV−REV). The construction of DIV−SEC as Herfindahl Hirschman Index in terms of sectoral diversification is described in Table S1 of a Supplementary file. Following prior studies using cross-country data (Le et al., 2020), income diversification is constructed as $DIV-REV=1-({\left(\frac{FIN}{TOTREV}\right)}^{2}+{\left(\frac{INV}{TOTREV}\right)}^{2}+{\left(\frac{FEE}{TOTREV}\right)}^{2}+{\left(\frac{OTHER}{TOTREV}\right)}^{2}$) where TOTREV=FIN+INV+FEE+OTHER; FIN, financing based income; INV, investment-based income; FEE, fee-based income; OTHER, other income. The value of DIV−REV ranges from 0 to 1, and its higher value means greater income diversification. Note that we also use Herfindahl Hirschman Index in terms of types of Shari'ah-compliant contracts. The coefficients on this index are positive though statistically not significant. The full results are available upon request.

Because Sukuk holding activities are a part of revenue diversification, we include DIV−REV and SUKU in a separate model. For control variables (X), we include cost-to-income ratio (bank efficiency), capitalization (the ratio of total capital to total assets), Islamic banking service coverage (the natural logarithm of Islamic bank branches).

As mentioned above, the global economy has faced a severe challenge caused by the COVID-19 turmoil. Although this crisis was not originated from the financial system, this health crisis has interrupted a whole economy by affecting both supply and demand sides. In response, governments around the world must implement several measures such as social distancing, lockdown. Consequently, this causes an increasing unemployment rate and business shutdown, which in turn affects bank performance. Along with the conventional banking systems, the Islamic banking systems will be affected by the COVID-19 outbreak. Eq. (1) is then modified to account for the impact of the COVID-19 pandemic.

(2) $${\text{π}}_{i,t}=\alpha_{0}+\alpha_{1}{\text{π}}_{i,t-1}+\alpha_{2}SUKU_{i,t}+\alpha_{3}DIV_{i,t}+\alpha_{4}X_{i,t}+\alpha_{5}CVD_{t}+\varepsilon_{i,t}$$

where CVD is a dummy variable that takes a value of 1 for 2020Q1-2020Q4 and 0 otherwise. Several studies consider the beginning of the year 2020 as the period of the COVID-19 pandemic (Demirgüç-Kunt et al., 2021; Elnahass et al., 2021) since the first infectious case was reported on 31 December 2019 in Wuhan and an emergency call of the World Health Organization on 11 March 2020 was then announced.^1^, ^2^ More specifically, Elnahass et al. (2021) use a dummy variable that takes a value of 1 for the first two quarters of the year 2020 to proxy for the impact of the COVID-19 pandemic due to data unavailability. We extend our analysis of the impact of the COVID-19 shock by examining the longer period from 2020Q1 to 2020Q4 as the spread of COVID-19 is still ongoing. It is important to note that the country-fixed effects are excluded in our regression because this exclusion ensures too many instruments occur within a small sample size of our study. Including country-fixed effects also prevents us from using a dummy variable, CVD as a proxy for the COVID-19 pandemic.^3^

### 3.2 Data

It is essential to mention that our data was gathered at the national or aggregate level. The data was primarily obtained from the Islamic Financial Services Board database (IFSB, 2021), covering 24 countries from 2013Q4 to 2020Q4. The banking systems included in our sample must have at least three consecutive years, especially to examine the effect of Sukuk holdings on the performance of the Islamic banking system. Hence, this arrives at an unbalanced sample of 23 countries between 2013Q4 and 2020Q4. The list of countries is presented in Table S2 of a Supplementary file.

Table 1 shows that the average return on assets (ROA) and the average return on equity (ROE) are 1.2% and 13.4%, respectively, with low volatility (i.e., low standard deviation). Regarding the diversification, the value of almost zero for the cases of DIV−SEC and DIV−REV argues that the banking system was the least diversified. For example, the Libyan Islamic banking system was most concentrated with fully financing to one sector (e.g., the wholesale and retail trade, and repair of motor vehicles and motorcycles) or with fully financing by Murābahah type of Shari'ah-compliant contract in 2018Q3. Similarly, the average ratio of Sukuk holdings to capital (SUKU) is 71.8%, with a low standard deviation. The minimum value of SUKU is 0, implying that there appear no Sukuk holdings in some Islamic banking systems in several quarters (e.g., Afghanistan's Islamic banking system in 2018Q2) due to the recent development of the Sukuk capital market in some countries.

**Table 1:** Descriptive statistics of variables used.

Variable	Obs	Mean	STD	Min	Max
ROA	510	0.012	0.012	-0.058	0.049
ROE	510	0.134	0.131	-0.484	0.950
DIV-SEC	454	0.737	0.145	0	0.921
DIV-REV	437	0.452	0.132	0.0002	0.788
CIR	494	0.718	1.032	0.119	11.139
CAP	539	0.118	0.088	0.020	0.768
LNBR	502	5.297	1.942	1.099	9.977
SUKU	539	0.718	0.971	0	7.572
CVD	538	0.128	0.335	0	1

Notes: ROA, return on assets; ROE, return on equity; DIV−SEC, Herfindahl Hirschman Index in terms of sectoral diversification; DIV−REV, Herfindahl Hirschman Index in terms of revenue diversification; CIR, cost-to-income ratio; CAP, the ratio of total capital to total assets; LNBR, the natural logarithm of Islamic bank branches; SUKU, the ratio of Sukuk holdings to capital; CVD, a dummy variable that takes a value of 1 for 2020Q1-2020Q4, and 0 otherwise; and Obs represents the number of observations.

Due to the worldwide impact of the COVID-19 pandemic, this has interrupted the global supply chain, and thus affecting both supply and demand of the world economy. This means that most countries have been affected by the COVID-19 turmoil directly and indirectly. Therefore, CVD equals 1 for all quarters from 2020Q1 to 2020Q4 for all Islamic banking systems since the announcement of the COVID-19 shock, and 0 for other quarters. As shown, the number of Islamic banking systems that suffered the impact of the COVID-19 pandemic was 12.8%.

**Figure 1:**
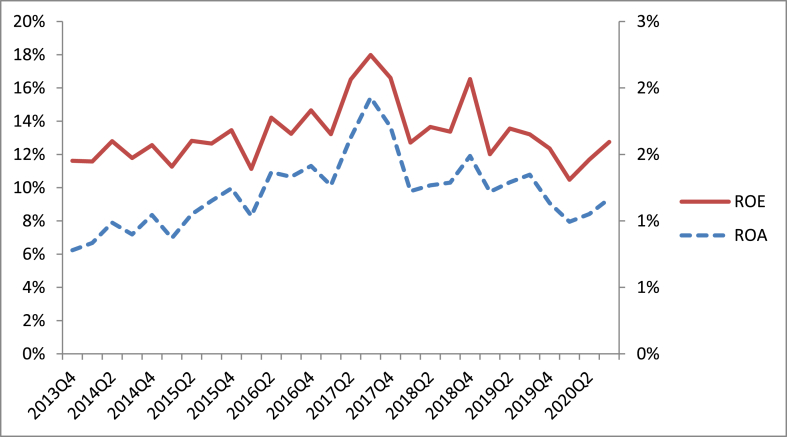
The evolution of bank profitability, ROA (right axis) and ROE (left axis), 2013Q4-2021Q1.

Figure 1 shows an increasing trend in both measures of bank profitability from 2013Q4 to 2017Q3 and then a decreasing trend in the latter period. Also, there appears to be a substantial reduction in ROA and ROE in the first quarter of 2020 due to the significant impact of the COVID-19 pandemic when implementing the social distancing policy measure and lockdown measures. However, a slight increase in ROA and ROE in the third quarter of 2020 may reflect a good sign of recovery. This is perhaps because the authorities may recognize that the strict social distancing policy may not be effective in the long term and gradually reactivate the production process after several lessons are drawn.

## 4 Empirical findings

Table 2 shows the correlation matrix of our variable used in this study. At first glance, there appears a positive impact of diversification (both measures, DIV−SEC and DIV−REV) on the profitability of the Islamic banking systems. The same is true in the case of Sukuk holdings (SUKU). Also, there is a negative impact of the COVID-19 pandemic on the profitability of Islamic banking systems. However, endogenous problems may arise as discussed above, and the use of the system GMM will be presented in the next section.

**Table 2:** Correlation matrix of variables used.

	ROA	ROE	DIV-SEC	DIV-REV	CIR	CAP	LNBR	SUKU	CVD
ROA	1								
ROE	0.767	1							
DIV-SEC	0.421	0.328	1						
DIV-REV	0.012	0.009	-0.125	1					
CIR	-0.594	-0.588	-0.488	0.121	1				
CAP	-0.462	-0.391	0.006	-0.057	0.06	1			
LNBR	0.336	0.518	-0.051	-0.028	-0.168	-0.667	1		
SUKU	0.12	0.001	-0.638	0.232	0.378	-0.411	0.440	1	
CVD	-0.027	-0.002	-0.017	0.042	0.076	-0.136	0.068	0.173	1

Notes: ROA, return on assets; ROE, return on equity; DIV−SEC, Herfindahl Hirschman Index in terms of sectoral diversification; DIV−REV, Herfindahl Hirschman Index in terms of revenue diversification; CIR, cost-to-income ratio; CAP, the ratio of total capital to total assets; LNBR, the natural logarithm of Islamic bank branches; SUKU, the ratio of Sukuk holdings to capital; CVD, a dummy variable that takes a value of 1 for 2020Q1-2020Q4, and 0 otherwise.

For diagnostic tests, the results show that the p-values of the Hansen test and the Arellano-Bond test for second-order autocorrelation are statistically not significant. This means that over-identifying restrictions do not exist, the moment conditions are fulfilled, and the instruments are justified. Furthermore, the coefficients of lagged measures of banking performance are significantly positive, implying that the system GMM is appropriate to use in this study.

For ease of exposition, we focus on our primary interest variables as presented in Table 3. The positive coefficients on DIV−SEC and DIV−REV imply that sectoral diversification of Shari'ah-compliant financing and revenue diversification tends to enhance the profitability of the Islamic banking systems. Therefore, hypotheses 1–2 cannot be rejected. This suggests that, in general, Islamic banking systems should increase their diversification degree in terms of either Shari'ah-compliant financing activities or their non-traditional activities (e.g. commission and fee income). This is significantly important to some Islamic banking systems (e.g. Libya, Afghanistan) where there appears a lower level of diversification. Molyneux and Yip (2013) demonstrate that Islamic banks may have the potential to capture diversification benefits compared to their conventional peers as they are relatively less diversified. More importantly, Islamic banks must follow Shari'ah law, so the net effect of diversification on their performance should be non-negative or even positive. Thus, any diseconomies and agency problems related to diversification should be reduced at a minimum (Magalhães and Al-Saad, 2013). Nonetheless, this somewhat supports the early findings of Smaoui et al. (2020), who suggest that Islamic rural banks in Indonesia tend to increase their margins when they have high loan diversification. Similarly, Chen et al. (2018) demonstrate that the benefits of diversification are more prevalent in the case of Islamic banks in three Asian countries.

**Table 3:** The results of our baseline models.

Π	ROA	ROA	ROA	ROA	ROE	ROE
*Π*_*t-1*_	0.48∗∗∗ (0.07)	0.373∗∗∗ (0.008)	0.484∗∗∗ (0.07)	0.445∗∗∗∗ (0.023)	0.619∗∗∗ (0.034)	0.427∗∗∗ (0.046)
*DIV-SEC*	0.082∗∗∗ (0.022)		0.065∗∗∗ (0.021)		0.089∗∗ (0.035)	
*DIV-REV*		0.036∗∗∗ (0.005)		0.018∗∗∗ (0.003)		0.182∗∗∗ (0.021)
*CIR*	-0.001 (0.002)	-0.004∗∗∗ (0.0002)	-0.002 (0.002)	-0.002∗∗∗ (0.0003)	-0.017∗∗∗ (0.001)	-0.012∗∗∗ (0.003)
*CAP*	-0.015 (0.011)	-0.007 (0.008)	-0.008 (0.013)	-0.016 (0.01)	0.102 (0.11)	-0.02 (0.066)
*LNBR*	0.000 (0.002)	0.004∗∗∗ (0.001)	0.002 (0.002)	0.002∗∗ (0.001)	0.016∗ (0.009)	0.023∗∗∗ (0.005)
*SUKU*	0.006∗∗∗ (0.002)		0.006∗∗∗ (0.002)		0.003 (0.005)	
CVD			-0.003∗∗∗ (0.001)	-0.001∗∗∗ (0.0002)	-0.012∗∗ (0.005)	-0.001 (0.003)
Constant	-0.056∗∗∗ (0.014)	-0.025∗∗∗ (0.006)	-0.051∗∗∗ (0.012)	-0.011 (0.007)	-0.1 (0.07)	-0.126∗∗∗ (0.023)
No. Obs	323	322	323	322	323	322
No. Instruments	13	21	14	17	26	17
AR1 (p-value)	0.042	0.088	0.038	0.08	0.097	0.114
AR2 (P-value)	0.948	0.548	0.806	0.569	0.303	0.337
Hansen test (p-value)	0.398	0.652	0.367	0.570	0.999	0.415

Notes: ROA, return on assets; ROE, return on equity; DIV−SEC, Herfindahl Hirschman Index in terms of sectoral diversification; DIV−REV, Herfindahl Hirschman Index in terms of revenue diversification; CIR, cost-to-income ratio; CAP, the ratio of total capital to total assets; LNBR, the natural logarithm of Islamic bank branches; SUKU, the ratio of Sukuk holdings to capital; CVD, a dummy variable that takes a value of 1 for 2020Q1-2020Q4, and 0 otherwise. Instrumental variables used in the GMM procedure, as proposed by Arellano and Bover (1995), are in italics. Robust standard errors are in parentheses. ∗, ∗∗, ∗∗∗Significance at the 10%, 5%, and 1% levels, respectively.

Furthermore, the coefficients of SUKU are positive and significant, thus hypothesis 3 is accepted. This implies that a greater level of Sukuk holdings can improve the profitability of the Islamic banking systems. This suggests that Sukuk holdings in the portfolios of the Islamic banking systems not only further promote the development of Sukuk financial markets and generate higher earnings as some of Sukuk issued are considered safe as qualified as Tier 1 (or Tier 2) capital (Smaoui et al., 2020). When observing Sukuk holdings as a part of income diversification, the positive coefficients on SUKU reemphasize such a positive effect of diversification on the profitability of Islamic banking systems. This may suggest the portfolio diversification benefits of Sukuk investment for investors (Najeeb et al., 2017). Indeed, the Sukuk sectors occupied 22.3% of the worth of the global Islamic financial services industry with its compound annual growth rate of 26% over the period 2004–2019 (IFSB, 2020).

Furthermore, the negative coefficients on CVD suggest that the Islamic banking systems are affected by the COVID-19 pandemic. As a result, hypothesis 4 cannot be rejected. Again, this reemphasizes the internationally broad impact of the COVID-19 turmoil on all banking systems. This somewhat supports the findings of Miah et al. (2021) using Islamic banks in Bangladesh. Nonetheless, this is consistent with Elnahass et al. (2021)'s findings the COVID-19 shock affects Islamic banks' profitability negatively. One may argue that the Islamic banking system may be less affected by this health crisis because of the nature of the constrained banking business model, characterized by profit and loss sharing and the forbidden speculative investments as well as the supervision of the board of directors and Sharia supervisor board (Elnahass et al., 2018). However, this health crisis has interrupted the global supply chain and a whole economy via both supply and demand sides. Due to the spread of Coronavirus, governments around the world have implemented several measures such as social distancing policy measures, lockdown, and business shutdown. This impact is more pronounced to microenterprises and small-and-medium enterprises as considered the main segment of the Islamic banking sector (Islamic Development Bank, 2020). Hence, the Islamic banking systems will suffer from the global health crisis, just as the conventional banking systems do (Elnahass et al., 2021).

The profitability of the Islamic banking systems is also negatively influenced by banking efficiency (CIR) and banking capitalization (CAP). Also, the positive coefficients on LNBR argue that the greater Islamic banking coverage would improve their profitability.

Due to the positive effect of diversification and negative impact of the COVID-19 pandemic, we further investigate the interaction effect of COVID-19 and diversification on the profitability of the Islamic banking systems. As shown in Table 4, the positive coefficients on DIV−REV∗CVD imply that hypothesis 5 is accepted. This finding suggests that revenue diversification may alleviate the adverse effect of the COVID-19 pandemic on Islamic banking profitability. Hence, the shift towards commission and fee income, other non-financing income may benefit the Islamic banking system during this health crisis. Past lessons show that the global banking system witnessed a decline in banks interest income (so-called financing income in Islamic banks) although the Islamic banking systems were less affected compared to the conventional ones (Beck et al., 2013). Since the global financial crisis 2007–09, Islamic banks have gradually engaged more in non-profit loss-sharing activities such as cost-plus (Murabaha), leasing (Ijarah), deferred payment sale (Bai’ Muajjal), forward sale (Bai’Salam), and contract manufacturing (Istisna). This shift has greater importance for the Islamic banking systems to overcome the negative impact of COVID-19 turmoil.

**Table 4:** The results of the interaction between COVID-19 and diversification.

Π	ROA	ROA	ROA	ROA	ROE	ROE	ROE
*Π*_*t-1*_	0.439∗∗ (0.182)	0.534∗∗∗ (0.057)	0.449∗∗∗ (0.06)	0.64∗∗∗ (0.15)	0.226∗∗∗ (0.059)	0.738∗∗∗ (0.034)	0.758∗∗∗ (0.053)
*DIV-SEC*	-0.003 (0.012)	0.047∗∗ (0.019)		0.018 (0.017)		0.199∗∗ (0.093)	0.038 (0.13)
*DIV-REV*			0.004 (0.009)		0.054 (0.093)		
*SUKU*	0.001 (0.002)	-0.004 (0.003)		-0.002 (0.002)		-0.019 (0.018)	0.026 (0.069)
CVD	-0.004 (0.008)	-0.005∗∗∗ (0.001)	-0.008∗∗∗ (0.03)	-0.026 (0.049)	-0.15∗∗∗ (0.035)	-0.026∗∗ (0.01)	-0.012 (0.219)
*DIV-SEC∗CVD*	0.006 (0.012)			0.026 (0.057)			-0.002 (0.269)
*DIV-REV∗CVD*			0.014∗∗ (0.005)		0.322∗∗∗ (0.061)		
*SUKU∗CVD*		0.004∗∗ (0.004)		0.005 (0.006)		0.021∗∗ (0.009	
Constant	0.051 (0.029)	-0.044∗∗∗ (0.013)	-0.025∗ (0.012)	-0.012 (0.016)	-0.08 (0.066)	-0.157∗ (0.085)	-0.07 (0.078)
Control variables	Yes	Yes	Yes	Yes	Yes	Yes	Yes
No. Obs	323	323	322	323	322	323	323
No. Instruments	23	16	26	18	20	16	16
AR1 (p-value)	0.082	0.05	0.079	0.083	0.204	0.093	0.086
AR2 (P-value)	0.725	0.708	0.626	0.810	0.727	0.600	0.256
Hansen test (p-value)	0.968	0.429	0.992	0.511	0.845	0.716	0.565

Notes: ROA, return on assets; ROE, return on equity; DIV−SEC, Herfindahl Hirschman Index in terms of sectoral diversification; DIV−REV, Herfindahl Hirschman Index in terms of revenue diversification; CIR, cost-to-income ratio; CAP, the ratio of total capital to total assets; LNBR, the natural logarithm of Islamic bank branches; SUKU, the ratio of Sukuk holdings to capital; CVD, a dummy variable that takes a value of 1 for 2020Q1-2020Q4, and 0 otherwise. Instrumental variables used in the GMM procedure, as proposed by Arellano and Bover (1995), are in italics. Robust standard errors are in parentheses. ∗, ∗∗, ∗∗∗Significance at the 10%, 5%, and 1% levels, respectively.

This positive effect of diversification during the COVID-19 shock is further confirmed by the positive coefficients of SUKU∗CVD. Thus, hypothesis 7 cannot be rejected. Again, Sukuk investments may generate higher returns for Islamic banks. Because Sukuk instruments are generally issued by governments or trusted enterprises to fund various projects, the risks of these financial products are relatively lower than other conventional ones in the markets. This is one of the unique features of Sukuk products. Therefore, Islamic Development Bank (2020) reported that the sovereign Sukuk market is expected to be less affected by the COVID-19 turmoil. For this reason, Sukuk markets have attracted much attention from bankers, policymakers and practitioners. However, the coefficients on *DIV-SEC∗CVD* are generally positive though statistically not significant, and thereby hypothesis 6 is rejected. In other words, we do not find any evidence that sectoral diversification of Shari'ah-compliant financing (DIV−SEC∗CVD) may reduce the impact of the COVID-19 on the performance of the Islamic banking systems. Although sectoral diversification, in general, may benefit Islamic banking systems, as shown above, this diversification strategy seems not to work during the impact of the unprecedented COVID-19 turmoil. As mentioned above, the COVID-19 pandemic has interrupted the operation and production processes of all firms regardless their sizes across various industries and thus affecting households. Therefore, the Islamic banking systems that have engaged in many sectors of Shari'ah-compliant financing may not gain diversification benefits during the COVID-19 period.

## 5 Conclusion

This study reexamined the relationship between diversification and Islamic banking systems' performance under the impact of the COVID-19 pandemic using a system GMM on the sample of 24 countries between 2013Q4 and 2020Q4. Our findings firstly unveil the benefits of diversification regarding sectoral diversification of Shari'ah-compliant financing and income diversification. Secondly, the results document an adverse impact of the COVID-19 shock on the Islamic banking systems, supporting the view of Elnahass et al. (2021) that the Islamic banking system will suffer this health crisis in the same way as the conventional banking system does. More importantly, the findings emphasize that income diversification may reduce the negative impact of the COVID-19 turmoil on the profitability of the Islamic banking systems. As income diversification may play a crucial role in helping the Islamic banking systems to deal with the COVID-19 pandemic, the findings indicate that Sukuk investment is considered an essential tool in this strategy. The participation of the Islamic banking systems in Sukuk markets would not only improve their performance but also enhance the liquidity of Sukuk markets, especially during the COVID-19 pandemic. Therefore, the authorities should encourage Islamic banks to engage more in both domestic and international Sukuk markets. Nonetheless, we do not find any evidence that sectoral diversification of Shari'ah-compliant financing may benefit the Islamic banking systems during this health crisis. This may reemphasize the vast implications of the COVID-19 shock on all sectors of Shari'ah-compliant financing that the Islamic banking systems have engaged. In the case of the ongoing spread of COVID-19 and its unknown variants in the future, the findings suggest that Islamic banking systems should put more attention on non-financing income, including the investment-based income (e.g., Sukuk and other Shari'ah-compliant securities) and other fee-based or even new types of income, as long as they are not violating the Shari'ah compliance.

However, our study may suffer some limitations. Due to data unavailability, this study used aggregated data of 24 countries from 2013Q4 to 2020Q4 to examine the relationship between different types of diversification strategies and the performance of Islamic banking systems. Perhaps, future research may use bank-level data to verify our findings. Furthermore, future studies may use alternative measures of COVID-19 as the use of a dummy variable (CVD) as a proxy of COVID-19 may suffer a disadvantage, as discussed above. If available, World Pandemic Uncertainty Index and the Discussion about Pandemic Index, or even the quarter infected cases and/or the quarter death cases, could be used. Last, it is acknowledged that different countries may impose different governments' responses to the COVID-19, such as social distancing measures, lockdown policies, containment and health policy measures, and economic stimulus packages. Thus, this may affect the economy's recovery across different countries, which further impact the relationship between diversification and the performance of Islamic banking systems during the COVID-19 pandemic. Hence, future work should consider governments' actions to the COVID-19 shock when observing the impact of diversification on the performance of Islamic banking systems.

## Declarations

### Author contribution statement

Tu DQ Le: Conceived and designed the experiments; Performed the experiments; Analyzed and interpreted the data; Contributed reagents, materials, analysis tools or data; Wrote the paper.

Tin H Ho: Conceived and designed the experiments; Wrote the paper.

Dat T Nguyen: Performed the experiments; Contributed reagents, materials, analysis tools or data.

Thanh Ngo: Analyzed and interpreted the data; Wrote the paper.

### Funding statement

This research was funded by the ​ University of Economics and Law, Vietnam National University, Ho Chi Minh, Vietnam.

### Data availability statement

Data will be made available on request.

### Declaration of interests statement

The authors declare no conflict of interest.

### Additional information

No additional information is available for this paper.

## Footnotes

1 COVID-19-China, World Health Organization https://www.who.int/emergencies/disease-outbreak-news/item/2020-DON233.

2 WHO Director-General's opening remarks at the media briefing on COVID-19–11 March 2020, World Health Organization, https://www.who.int/director-general/speeches/detail/who-director-general-s-opening-remarks-at-the-media-briefing-on-covid-19---11-march-2020.

3 Note that the data on country-specific characteristics (e.g., banking competition, market concentration, banking regulation, and other institutional features are not available during the impact of COVID-19 as well as on a quarterly basis. The use of CVD as a dummy variable may suffer a drawback since not all countries were immediately affected by the COVID-19 pandemic and this is supposed to be a lagged effect of COVID-19 shock among countries. The use of the World Pandemic Uncertainty Index and the Discussion about Pandemic Index may better capture the impact of the COVID-19 shock on each economy. However, these indices are missing in most countries in our sample.
