# Supplementary material for: A cross-country analysis on diversification, Sukuk investment, and the performance of Islamic banking systems under the COVID-19 pandemic
Source: Heliyon. 2022 Mar 12;8(3):e09106. doi: 10.1016/j.heliyon.2022.e09106 (PMC8917644; doi:10.1016/j.heliyon.2022.e09106)
Supplement: Supplementary file [file mmc1.docx]

# Supplementary file

**Table S1** Sectoral diversification of Shari'ah-compliant financing

| Agriculture; forestry; hunting; and fishing | Transportation and storage | Public administration and defense; and compulsory social security |
| --- | --- | --- |
| Mining and quarrying | Accommodation and food service activities (general goods) | Machinery and vehicle |
| Manufacturing | Information and communication | Human health and social work activities |
| Electricity; gas; steam; and air-conditioning supply (fuel, petroleum) | Financial and insurance activities | Arts; entertainment; and recreation |
| Water supply; sewerage and waste management | Real estate activities (assets acquired for leasing) | Other service activities (export business) |
| Construction | Professional; scientific; and technical activities | Activities of households as employers |
| Wholesale and retail trade; repair of motor vehicles and motorcycles | Administrative and support service activities | Activities of extraterritorial organizations and bodies |

Source: Adapted from IFSB (2021)

According to the Islamic Financial Services Board classification, Shari'ah-compliant financing can be distributed into 21 sectors, as shown in Table S1. Therefore, the value of Shari'ah-compliant financing is the sum value of each category (21 in total). Similar to the construction of $\text{DIV-REV}$, the Herfindahl Hirschman Index in terms of sectoral diversification ($\text{DIV-SEC}$) is constructed. Again, a higher value of $\text{DIV-SEC}$ implies greater sectoral diversification.

Table S2 The list of countries in the sample

| Afghanistan | Iran | Malaysia | Saudi Arabia |
| --- | --- | --- | --- |
| Bahrain | Jordan | Nigeria | Sudan |
| Bangladesh | Kazakhstan | Oman | Turkey |
| Brunei Darussalam | Kuwait | Pakistan | United Arab Emirates |
| Egypt | Lebanon | Palestine | United Kingdom |
| Indonesia | Libya | Qatar |  |
